# Reactive oxygen species and peroxynitrite in acetaminophen-induced liver injury: Lipid peroxidation and ferroptosis-like cell death

**DOI:** 10.70401/fos.2025.0007

**Published:** 2025-12-19

**Authors:** Hartmut Jaeschke, Anup Ramachandran

**Affiliations:** Department of Pharmacology, Toxicology and Therapeutics, University of Kansas Medical Center, Kansas, KS 66160, USA.

**Keywords:** Acetaminophen, drug-induced liver injury, acute liver failure, ferroptosis, oxidant stress, lipid peroxidation

## Abstract

Acetaminophen (APAP) overdose is a clinically relevant model of drug hepatotoxicity and acute liver failure. After decades of research, many aspects of the mechanism of APAP-induced liver injury are well established. These include the cytochrome P450 2E1-mediated formation of a reactive metabolite, hepatic glutathione depletion, mitochondrial protein adduct formation with oxidant stress and peroxynitrite formation, iron-catalyzed protein nitration in mitochondria, the opening of the mitochondrial permeability transition pore, and release of mitochondrial intermembrane proteins including endonuclease G, which translocate to the nucleus and cause DNA fragmentation, the final step of cell necrosis signaling. However, the mode of cell death remains controversial, as there are many overlaps with apoptosis, necroptosis, and pyroptosis. More recently, ferroptosis has come into focus as a popular cell death mode, creating a new controversial topic. The current review addresses some of the similarities and differences between ferroptosis and APAP-induced necrosis. For example, there is extensive glutathione depletion, but glutathione peroxidase 4 activity is not impaired; there is oxidant stress, but superoxide is used to form peroxynitrite; and there is evidence for an important role of ferrous iron as a catalyst for protein nitration. Moreover, lipid peroxidation is very limited, and excess Vitamin E does not protect. However, cotreatment of an APAP overdose with exogenous ferrous iron can induce extensive lipid peroxidation and switch the mode of cell death. Thus, APAP hepatotoxicity does not involve ferroptosis under normal, clinically relevant conditions, but a change in co-ingested supplements can trigger a switch to ferroptosis-like cell death.

## Graphical Abstract

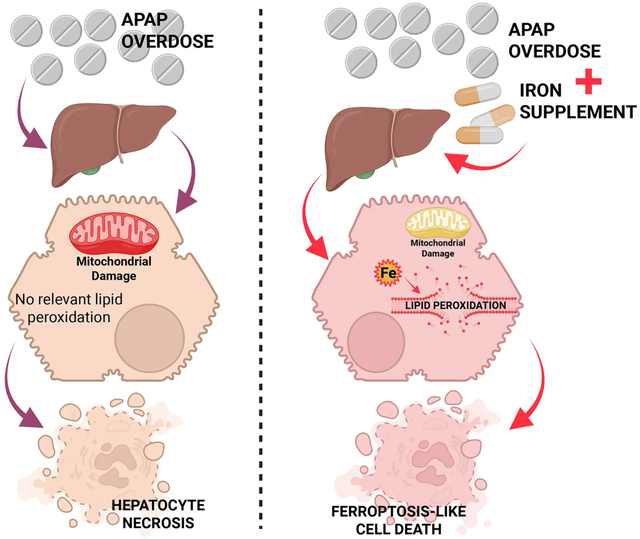

## 1. Introduction

Acetaminophen (APAP, paracetamol) is a safe and effective analgesic and antipyretic when used at therapeutic doses of less than 4 g/day. However, an overdose can cause liver injury or even acute liver failure^[1–3]^. In fact, APAP toxicity is the leading cause of acute liver failure in the Western world^[2]^. Therefore, it is clinically important to identify therapeutic targets and develop intervention strategies against APAP toxicity and acute liver failure^[4]^.

However, progress in this area depends on a solid understanding of the molecular mechanisms of APAP-induced cell death in experimental models and humans^[5]^. The first critical insight into the mechanisms of APAP hepatotoxicity was provided by scientists from the National Institutes of Health in 1973^[6–8]^. They reported a cytochrome P450-mediated formation of a reactive metabolite, now identified as N-acetyl-p-benzoquinone imine (NAPQI)^[9]^, which can be detoxified by glutathione (GSH)^[7,8]^. However, an overdose exhausts the hepatic GSH stores, and the remaining reactive metabolites covalently bind to sulfhydryl groups of cysteine residues in proteins^[10]^. The formation of protein adducts correlated with liver injury, establishing protein binding as the initial hypothesis of APAP toxicity^[6–8]^. A few years after these publications, a competing hypothesis was proposed, suggesting that the formation of reactive oxygen species (ROS) during the metabolism of APAP by cytochrome P450 enzymes results in extensive lipid peroxidation (LPO), which is responsible for cell death^[11,12]^. These competing hypotheses led to a substantial debate regarding the role of ROS and LPO in the pathophysiology. This controversial discussion was revived with the more recent introduction of ferroptosis as a unique mode of cell death^[13]^. This review will evaluate key elements of the necrotic cell death process in APAP hepatotoxicity, including ROS and peroxynitrite formation, lipid peroxidation, and the role of iron, to answer the question of whether ferroptosis-like cell death occurs after an APAP overdose and under what clinical circumstances this could be relevant.

## 2. Reactive Oxygen Species

To understand the role of oxidant stress in APAP toxicity, it is essential to identify the specific reactive oxygen species involved, their sources, and the detailed mechanisms by which these ROS contribute to the injury process.

### 2.1 ROS and cytochrome P450 enzymes

It was first hypothesized that the metabolism of APAP by cytochrome P450 generates ROS, which serve as initiators of LPO^[12]^. This conclusion was based on in vitro experiments showing both superoxide and hydrogen peroxide formation during microsomal drug metabolism^[14]^, and the modulation of LPO and injury in vivo with P450 inducers and inhibitors^[12]^. However, measurement of glutathione disulfide (GSSG), a product generated during the reduction of hydrogen peroxide by glutathione peroxidase, did not support the hypothesis that ROS are generated during the metabolism of APAP in rats or mice^[15,16]^.

### 2.2 ROS and xanthine oxidase

In the 1980s, it was postulated that xanthine oxidase (XO), converted from the dehydrogenase, is a critical source of superoxide during ischemia-reperfusion injury^[17]^. Although an increase in XO activities was detected during APAP toxicity and the XO inhibitor allopurinol was protective^[18,19]^, a dose-response experiment with allopurinol showed that the beneficial effect of allopurinol requires an almost 10-fold higher dose than is needed for complete inhibition of XO^[18]^. These results indicated that XO is not a relevant source of oxidant stress in this model. Follow-up experiments showed that allopurinol is metabolized by aldehyde dehydrogenase, leading to a preconditioning effect with induction of metallothionein^[20]^, which protects against APAP toxicity by scavenging NAPQI^[21]^.

### 2.3 ROS and mitochondria

In contrast to these negative data, a time course of GSSG formation in the liver revealed a late increase (≥ 6 h after APAP)^[18]^. Interestingly, this GSSG appeared to be generated mainly in mitochondria, implicating electron transport chain leakage as a major source of ROS^[18,22]^. The trigger for the electron leak is protein adduct formation in the mitochondria^[23,24]^. Interestingly, selective transfection studies with Cyp2E1, the key cytochrome P450 enzyme responsible for the oxidative metabolism of APAP^[25,26]^, demonstrated that mitochondrial oxidant stress and cell death correlated with mitochondrial Cyp2E1 expression^[27]^. This was supported by recent studies showing that after exposure to an APAP overdose, hepatocytes, which express Cyp2E1 in mitochondria and in the ER, show protein adducts in both organelles but die through a mitochondria dysfunction-dependent necrosis^[28]^. In contrast, in proximal tubular cells of the kidney, Cyp2E1 is only expressed in the ER, which triggers adduct formation only in the ER, resulting in ER stress-mediated apoptosis^[28]^. Together, these data strongly support the hypothesis that in the liver, mitochondrial Cyp2E1 generates the NAPQI responsible for protein adduct formation and the electron leak in the mitochondria. However, a more detailed analysis of the mitochondrial oxidant stress showed a biphasic response^[29]^. An initial electron leak from complex III of the electron transport chain results in the release of free radicals into the intermembrane space and cytosol without influencing mitochondrial function^[29]^. In contrast, a later, amplified oxidant stress is mediated mainly by complex I, directed exclusively toward the mitochondrial matrix^[29]^.

Although oxidant stress may be harmful, extensive cellular defense mechanisms, including superoxide dismutases, glutathione peroxidases, catalase, and others, can limit the impact of ROS production^[30]^. However, peroxynitrite, an aggressive oxidant and nitrating species, was recognized to be generated during APAP toxicity^[31]^. Peroxynitrite is formed by the spontaneous combination of two radicals, the superoxide radical and the nitric oxide radical^[32]^. Because superoxide anions are generated in the mitochondrial matrix and cannot cross membranes, it was not surprising to find nitrotyrosine protein adducts mainly in mitochondria after an APAP overdose^[33]^. Although peroxynitrite can be effectively scavenged with GSH^[34]^, the severe depletion of cellular GSH after APAP leaves mitochondria without antioxidant defenses long enough to induce necrotic cell death. Delayed treatment with GSH or N-acetylcysteine accelerates the resynthesis of hepatic GSH levels and the restoration of the capacity to scavenge peroxynitrite, which attenuates APAP-induced liver injury^[34–36]^. Further experiments documented the multiple functions of cysteine supply. During APAP metabolism, GSH scavenges NAPQI, and after the metabolism phase, it scavenges peroxynitrite. In addition, any surplus of cysteine can be degraded and used as Krebs cycle intermediates to support mitochondrial energetics^[36]^. However, the most direct evidence for the pathophysiological role of mitochondrial peroxynitrite was obtained with partial SOD2-deficient mice, which showed an increase in nitrotyrosine staining and a dramatic aggravation of liver injury^[37,38]^. On the other hand, post-treatment with mitochondria-targeted SOD mimetics (Mito-Tempo, mitoquinone) eliminated nitrotyrosine staining and APAP hepatotoxicity^[39,40]^. In addition, peroxynitrite can inactivate SOD2 in the mitochondria^[41,42]^. Together, these data indicate that SOD2 in mitochondria is critical for limiting peroxynitrite formation under these conditions, suggesting that this reactive nitrogen species is the key oxidant and nitrating compound in the pathophysiology.

Although mitochondria are clearly established as the source of superoxide, the nitric oxide synthase (NOS) responsible for NO formation remains unclear. Originally, the inducible NOS (iNOS) was proposed as the source of NO^[43,44]^; however, the relevance was questioned when peroxynitrite-induced liver injury after an APAP overdose was found to be independent of iNOS induction^[22]^ and iNOS inhibitors did not protect^[45]^. In contrast, an inhibitor of the neuronal NOS (nNOS) was shown to reduce APAP-induced cell death in primary mouse hepatocytes^[46]^. Additionally, nNOS-deficient mice exhibited reduced nitrotyrosine staining and hepatic necrosis^[47]^. The hypothesis that nNOS might be the dominant producer of NO was indirectly supported by the protective effect of a calmodulin antagonist that inhibits Ca^2+^-induced nNOS activation in this model^[48]^. However, the location of nNOS in the liver remains elusive, as no NOS activity was detected in liver mitochondria^[49]^. As a gas, NO can diffuse through membranes and can act on neighboring cells. Therefore, a more distant site of formation may need to be considered. To this effect, nNOS-positive nerve fibers were detected in the liver^[50]^. However, more studies are required to conclusively identify the location of nNOS in the liver and determine under which conditions various NOS enzymes may contribute to APAP-induced liver injury.

**Figure 1.**
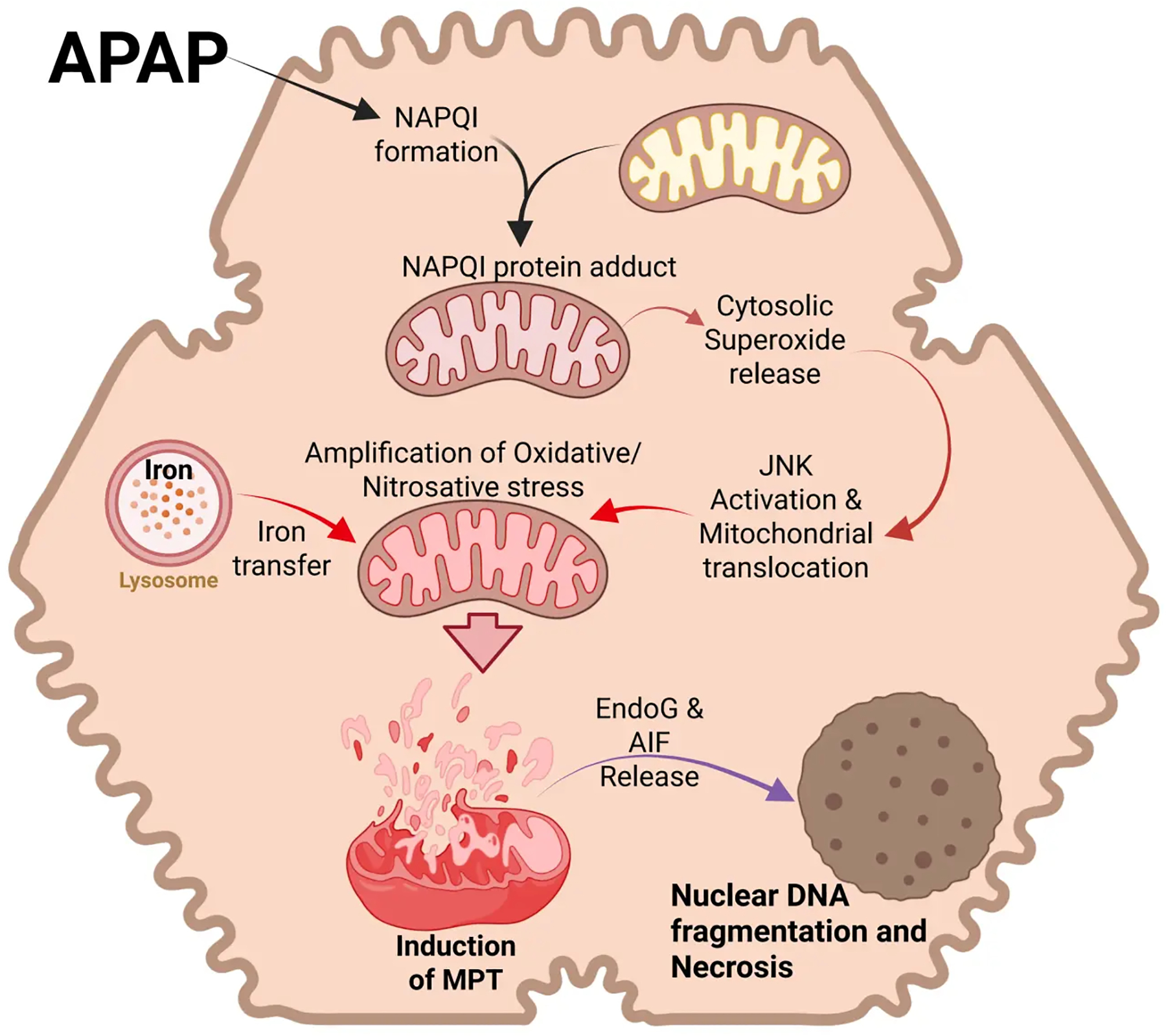
Cell signaling in APAP hepatotoxicity. APAP overdose induces excessive formation of the reactive metabolite NAPQI, which forms mitochondrial protein adducts. While this initially induces superoxide release from mitochondria to cytosol, the subsequent activation of the MAP kinase JNK and its translocation to mitochondria amplifies mitochondrial oxidative and nitrosative stress, facilitated by lysosomal iron transfer. This results in the mitochondrial permeability transition and release of mitochondrial proteins such as Endo G and AIF. These translocate to the nucleus to induce DNA fragmentation and cell necrosis. Created in BioRender.com. APAP: acetaminophen; NAPQI: N-acetyl-p-benzoquinone imine; MAP kinase: mitogen-activated protein kinase; JNK: c-Jun N-terminal kinase; Endo G: endonuclease G; AIF: apoptosis-inducing factor.

Although there is early evidence for mitochondrial oxidant stress^[29]^, the mitochondrial membrane permeability transition pore (MPTP) opening and cell death occur several hours later^[51,52]^. This led to the discovery of an oxidant stress amplification cycle involving c-Jun N-terminal kinase (JNK)^[53]^. The initial oxidant stress in the cytosol caused by the release of superoxide from the mitochondria^[29]^ leads to activation of a mitogen-activated protein kinase cascade. The ASK1-thioredoxin complex is redox sensitive, i.e., oxidation of thioredoxin liberates and activates ASK1^[54]^, leading to phosphorylation of MKK4^[55]^ and finally JNK^[56]^. Importantly, P-JNK translocates to the mitochondria and binds to the anchor protein Sab on the outer mitochondrial membrane^[56,57]^. This triggers inactivation of mitochondrial Src and causes a further impairment of the electron transport chain and an amplified release of electrons^[58]^, especially from complex I, into the mitochondrial matrix^[29]^. This amplified oxidant stress and peroxynitrite formation can trigger the MPTP opening^[45]^ (Figure 1). Hence, ASK1 and especially JNK inhibitors are highly effective in preventing APAP-induced mitochondrial dysfunction, oxidant stress, and cell death in murine and human hepatocytes^[56,59,60]^ and in vivo^[45,59,61,62]^.

## 3. Role of Iron in APAP-Induced Liver Injury

### 3.1 Iron and acetaminophen-induced cell death

Ferrous iron is considered a catalyst for the Fenton reaction, which generates hydroxyl radicals as initiators of LPO^[63,64]^. However, early studies with the iron chelator deferoxamine showed a reduction in the limited LPO but no effect on the liver injury after an APAP overdose^[65]^. Subsequent publications reported variable protection with iron chelators in this model^[66,67]^. Because no relevant LPO was observed, the interest in the role of iron was limited for some time. This changed when it was reported that the translocation of lysosomal iron to mitochondria during peroxide-mediated oxidant stress implicated iron in playing a critical role in the MPTP opening and cell death of cultured hepatocytes^[68]^. A similar mechanism was also discovered during APAP-induced cell death in hepatocytes, where lysosomal ferrous iron was released into the cytosol and then taken up into mitochondria^[69]^ (Figure 1). This conclusion was based on experiments with starch-desferal, a selective iron chelator for lysosomes, which eliminated iron uptake into the mitochondria and the MPTP opening^[69]^. Minocycline, an inhibitor of the mitochondrial electrogenic Ca^2+^ uniporter (MCU), blocked iron uptake into mitochondria and necrotic cell death both in vitro^[70]^ and in vivo^[71]^. The same effect was observed in global and hepatocyte-specific MCU knockout mice^[72]^. Together, these observations provided solid evidence for an important role of ferrous iron in APAP-induced cell death.

### 3.2 Iron and protein nitration

Despite the strong experimental support for iron as an important mediator of the injury process, this hypothesis does not align with the traditional role of ferrous iron in the Fenton reaction^[63,64]^ and the very limited lipid peroxidation observed in this model^[65,73]^. Furthermore, the hypothesis is inconsistent with the critical role of peroxynitrite in mitochondrial dysfunction and APAP-induced liver injury^[37,39]^. However, studies evaluating the mechanism of protein nitration by peroxynitrite have documented an essential role of transition metals, such as iron, in the reaction mechanism^[74]^. In support of this hypothesis, it was shown that the iron chelator deferoxamine and the MCU inhibitor minocycline effectively prevented protein nitration and APAP-induced liver injury in the absence of LPO^[75]^. Additionally, ferritinophagy was identified as the key mechanism underlying lysosomal iron release and mitochondrial protein nitration^[76]^. Thus, ferrous iron is a critical mediator in the pathophysiology of APAP-induced cell death through its uptake into mitochondria, which promotes the injury process by facilitating protein nitration, not by supporting Fenton reaction and lipid peroxidation under normal conditions.

## 4. Role of Lipid Peroxidation in APAP Hepatotoxicity

### 4.1 Lipid peroxidation as a mechanism of acetaminophen-induced cell death

The potential role of LPO in APAP toxicity has been controversially discussed for decades. In the late 1970s, it was first shown that an APAP overdose triggered massive LPO in the liver, measured by ethane and pentane exhalation, and severe liver injury with high mortality in a murine model^[11]^. LPO and liver injury could be prevented by cytochrome P450 inhibitors^[12]^ and by N-acetylcysteine or GSH treatment^[77]^. Similar massive LPO and liver injury were observed with another hepatotoxin, allyl alcohol, in the same mice^[78]^. In this model, iron chelation and vitamin E pretreatment eliminated LPO and liver injury^[78,79]^. However, other groups were not able to reproduce these findings. LPO was moderate, and the injury could not be prevented by iron chelation or vitamin E treatment^[65,73]^. It seemed difficult to reconcile these contradictory findings. However, it was not considered at that time that the animals used in these experiments were fed a diet high in unsaturated fatty acids (soybean oil) and low in vitamin E^[11,12,77–79]^. This led to a significant increase in the incorporation of arachidonic acid (20:4) and docosahexaenoic acid (22:6) in liver membranes compared to animals on a normal diet^[78]^. Because these were also the polyunsaturated fatty acids that were lost during LPO^[78]^, these observations suggest that this diet substantially enhanced the susceptibility to LPO and liver injury. However, what was intended to amplify LPO to more accurately measure LPO parameters, such as ethane and pentane exhalation in individual mice, resulted in a total switch of the injury mechanisms from protein adduct-mediated necrosis to LPO. This was only recognized years after the original controversy in the 1980s^[80]^. Interestingly, the data generated with the diet high in polyunsaturated fatty acids (PUFAs) and low in vitamin E or with ferrous iron co-treatment could be used as positive controls, i.e., models where LPO is the dominant injury mechanism. LPO parameters, including ethane and pentane exhalation, and hepatic levels of malondialdehyde (MDA), 4-hydroxynonenal (4HNE) or hydroxyeicosatetraenoic acids (HETEs) increase by 1,000 to 5,000 % above baseline^[12,73,75,81]^. Thus, under normal conditions, when these LPO parameters do not increase at all or at most 50–200% above controls, LPO is not a relevant injury mechanism in the APAP hepatotoxicity model.

### 4.2 Exogenous iron and lipid peroxidation after an APAP overdose

**Figure 2.**
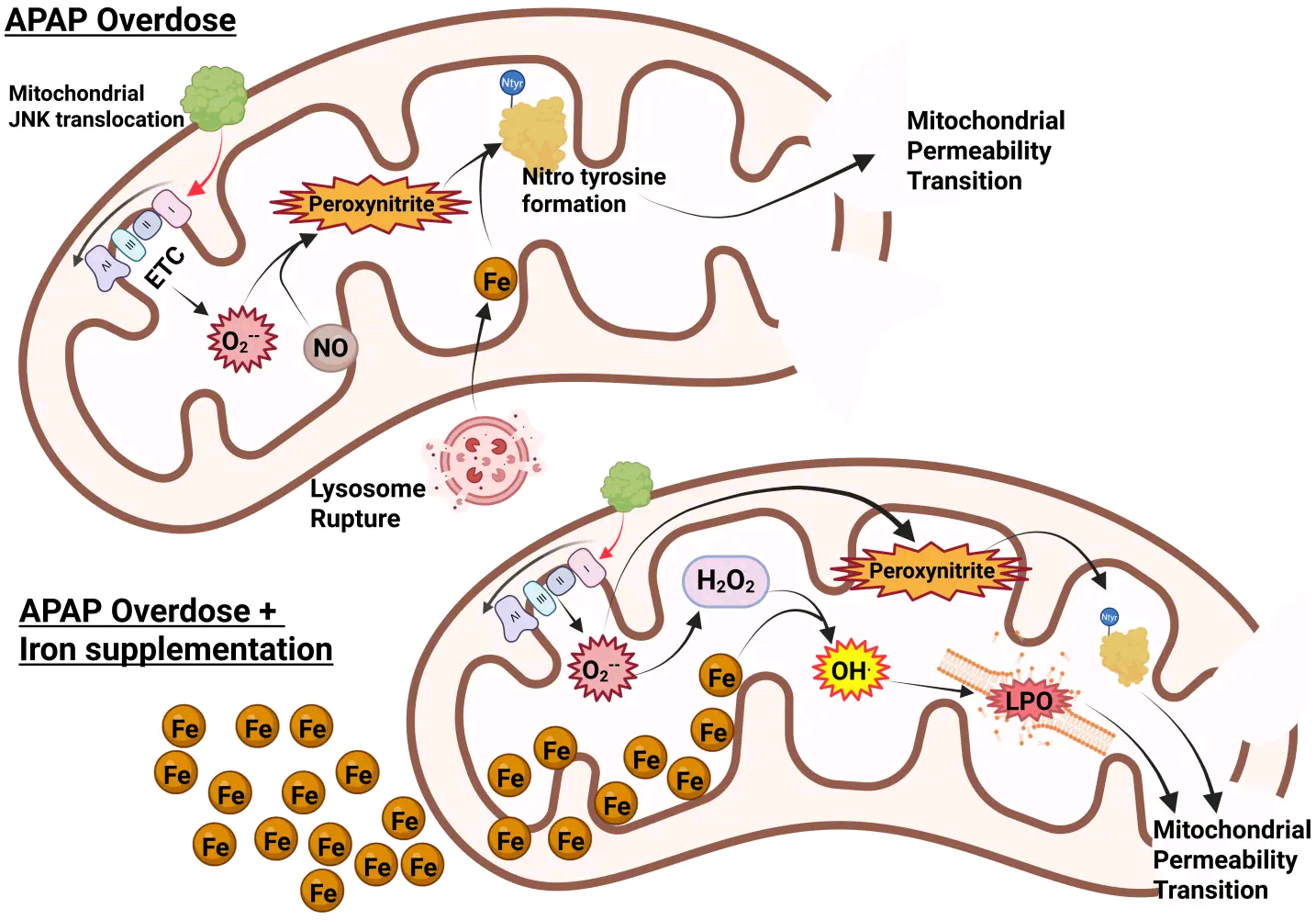
Role of iron in APAP-induced mitochondrial damage. Typically, after an APAP overdose, mitochondrial JNK translocation amplifies mitochondrial superoxide generation through ETC dysfunction. This reacts with nitric oxide to produce peroxynitrite, which requires iron (released from lysosomes) to catalyze the formation of nitrotyrosine adducts on proteins, leading to induction of the MPT and ultimately cell necrosis. With significant iron loading, however, it facilitates the Fenton reaction, producing hydroxyl radicals and lipid peroxidation to further amplify the MPT and then necrosis. Created in BioRender.com. APAP: acetaminophen; JNK: c-Jun N-terminal kinase; ETC: electron transport chain; MPT: mitochondrial permeability transition.

In Western countries, where APAP overdose is the most frequent cause of acute liver failure, vitamin E deficiency is not a relevant clinical problem. However, co-ingestion of an overdose of APAP with iron supplements can occur^[82,83]^. Thus, when mice are co-treated with a moderate dose of ferrous iron and an APAP overdose, liver injury is severely aggravated^[75]^. In addition, protein nitration is significantly enhanced, accompanied by a dramatic increase in LPO parameters (MDA, 4HNE, HETEs)^[75,81]^ (Figure 2). However, most importantly, an intervention that limits mitochondrial peroxynitrite formation (Mito-TEMPO), which is highly effective under normal conditions^[39]^, is no longer protective in the presence of ferrous iron^[81]^. Likewise, delayed treatment with the clinical antidote N-acetylcysteine, which supports GSH synthesis and the scavenging of peroxynitrite in the mitochondria^[36]^, is no longer protective in the model with iron co-treatment^[81]^. These data demonstrate that co-treatment with a moderate dose of ferrous iron and an APAP overdose again switches the mechanism of cell death from mitochondria dysfunction-centered necrosis to LPO as the dominant mechanism of cell death^[81]^. This is clinically important, as under these conditions, the standard of care, N-acetylcysteine, is no longer effective. Only an iron chelator can interrupt this process^[75]^. Interestingly, because N-acetylcysteine is highly effective in most APAP overdose patients^[84,85]^ and in human hepatocytes^[60]^, these data also argue against LPO as a relevant mechanism of APAP-induced cell death in patients under normal conditions in the absence of exogenous ferrous iron.

### 4.3 Mitochondrial aldehydes and APAP hepatotoxicity

**Figure 3.**
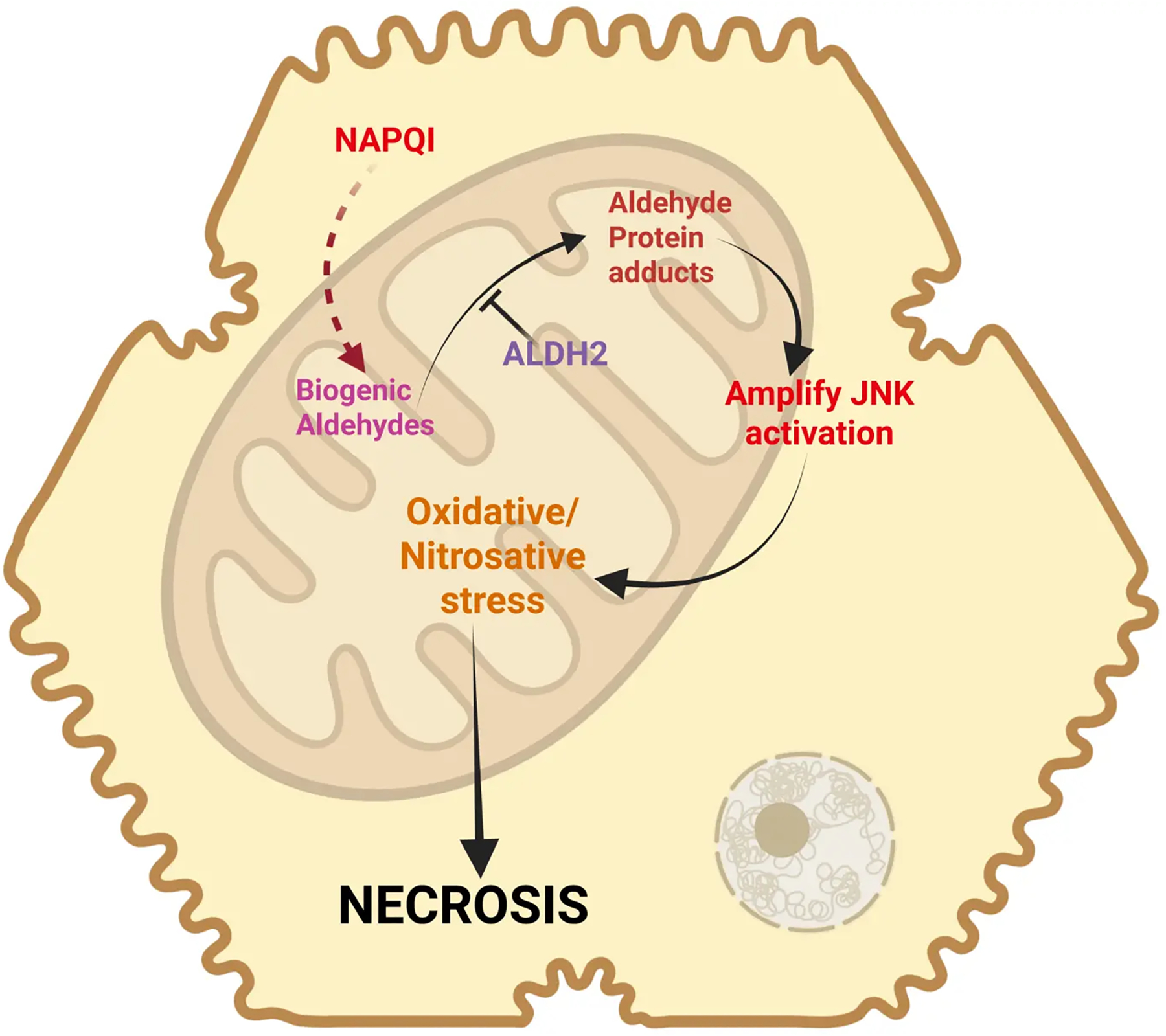
Biogenic aldehydes in APAP toxicity. Recent research has identified the generation of several biogenic aldehydes, which can be metabolized by ALDH2 to prevent downstream events. In its absence, these species can form aldehyde protein adducts and amplify JNK activation. This increase in JNK activation would enhance oxidative and nitrosative stress within mitochondria to amplify dysfunction and induce cell necrosis. Created in BioRender.com. APAP: acetaminophen; JNK: c-Jun N-terminal kinase; ALDH2: aldehyde dehydrogenase 2; NAPQI: N-acetyl-p-benzoquinone imine.

Although the absence of relevant LPO and the importance of mitochondrial peroxynitrite formation appear to be solidly supported by numerous mechanistic studies, more recently, yet another somewhat ignored aspect came into focus. Induction of the mitochondrial enzyme aldehyde dehydrogenase 2 (ALDH2) by Alda-1 resulted in the reduction of 4HNE formation, attenuated the MPTP opening, and reduced liver injury after an APAP overdose^[86]^. Protein adducts formation and JNK activation were not affected, indicating that the protective mechanism was at the level of mitochondria^[86]^. These data are consistent with the aggravation of APAP-induced liver injury in ALDH2-deficient mice^[87]^. Furthermore, SIRT3 can deacetylate lysine residues on ALDH2, thereby opening up targets for NAPQI binding and inactivation of the enzyme^[88]^. In SIRT3-deficient mice, ALDH2 activity is preserved, and APAP-induced liver injury is attenuated^[88]^. On the other hand, SIRT5 can de-succinylate lysine residues, especially lysine 385, which preserves the function of ALDH2, reduces mitochondrial oxidant stress, and protects against APAP toxicity^[89]^. Together, these observations strongly suggest that ALDH2 is an important enzyme in APAP hepatotoxicity. However, the overall low levels of prominent aldehydes such as MDA and 4HNE raise questions about the mechanism of this effect. Interestingly, a recent study provides new insights into this dilemma^[90]^. These investigators identified low levels of 74 free biogenic aldehydes in mouse livers after an APAP overdose^[90]^. Most importantly, 47 of these aldehydes can bind to proteins, with peak levels occurring 2–3 hours after APAP administration^[90]^. Thus, the time course of NAPQI protein adducts formation correlates well with that of the aldehyde protein adducts, both of which can be eliminated by a Cyp2E1 inhibitor^[90–92]^. In addition, the reduction of protein-bound aldehydes and reduced liver injury in animals treated with the ALDH2 inducer Alda-1 or an aldehyde scavenger (N-acetyl-l-lysine) suggests a critical role of these aldehydes in APAP-induced cell death^[90]^. These aldehydes can also induce JNK activation^[90]^. Thus, the formation of a large number of aldehydes at low levels can contribute to the toxicity by forming a variety of protein adducts in mitochondria and by promoting mitochondrial oxidant stress through JNK activation (Figure 3). The fact that reducing the levels of these aldehydes by ALDH2 induction or N-acetyl-L-lysine treatment is overall protective indicates that mitochondrial dysfunction and the MPTP opening involve both peroxynitrite-mediated mitochondrial protein nitration and protein binding of these aldehydes. This means that both toxicological events may be additive and may be necessary to trigger mitochondrial dysfunction and cell necrosis. Importantly, the aldehyde formation, although catalyzed by the Fenton-type LPO mechanism, does not cause direct membrane rupture, as would be expected during ferroptosis, but mainly supports the traditional mitochondrial injury pathway.

## 5. Ferroptosis in APAP-induced Liver Injury

### 5.1 Acetaminophen-induced ferroptosis under normal conditions

The term “ferroptosis” was introduced in a report on a distinct mode of cell death induced by erastin in an NRAS mutant HT-1080 fibrosarcoma cell line. Key features of this new cell death include “the loss of lipid peroxide repair capacity by the phospholipid glutathione hydroperoxidase GPx4, the availability of redox-active iron, and oxidation of polyunsaturated fatty acid—containing phospholipids^[93]^”. Thus, superficially, APAP-induced cell death fits most of these characteristics of ferroptosis. There is extensive GSH depletion due to the scavenging of NAPQI^[91]^, which would be expected to impair GPx4. However, during the early phase of drug metabolism and GSH depletion, there is no evidence for oxidant stress in vivo or in primary hepatocytes^[16,51]^, which would require GPx4 activity. In contrast, during the injury phase, when there is oxidant stress, the formation of HETEs as a direct indicator of GPx4 activity does not appear to be impaired^[81]^. The reason for this effect may be that even a 90% depletion of hepatic GSH levels, as observed after an APAP overdose^[91]^, still leaves enough GSH (0.5–1.0 mM) as a cofactor for this enzyme activity. Besides, hepatic GSH levels recover at least in part during the later phase of the injury^[91]^, which makes it even less likely that GSH levels are the limiting factor in lipid peroxide detoxification. Furthermore, the dependence of cell death on ferrous iron does not mean that a Fenton-type reaction is involved^[63,64]^. In contrast, there is strong support for the hypothesis that ferrous iron in mitochondria is essential for protein nitration by peroxynitrite^[75]^. Finally, LPO of PUFAs can occur^[94]^; however, LPO is quantitatively very limited, as indicated by hepatic levels of 4HNE, MDA, and HETEs, and ethane and pentane exhalation^[65,73,75,81,95,96]^. These observations are consistent with the lack of protection by ferroptosis inhibitors^[97]^ and by excess hepatic vitamin E^[73]^. Thus, despite several overlapping features, the cell death caused by an APAP overdose does not meet the definition of ferroptosis under normal circumstances.

### 5.2 Acetaminophen-induced ferroptosis in the literature

Despite the clear mechanistic evidence against ferroptosis in APAP-induced liver injury in mice, and indirectly also in humans, there is an exponential rise in published studies that claim ferroptotic cell death in this model. What is the reason for this development? These studies can be divided into 2 categories: The first class is studies that assess the beneficial effects of therapeutic interventions and use ferroptosis as a fashionable mode of cell death to explain protective mechanisms such as antioxidant activity and anti-inflammatory properties^[98–100]^. The conclusions are generally only based on correlations between liver injury and protection and the modulation of assumed ferroptosis parameters, e.g., GPx4 and acyl–coenzyme A synthetase long-chain family member 4 (ACSL4) protein levels, MDA, and 4-HNE. However, these are just correlations without established causality^[98–100]^, and as was recently pointed out, there are no specific biomarkers for ferroptosis^[101]^. In addition, no alternative mechanisms or interpretations of their data are discussed. Overall, it appears that the mode of cell death is of limited relevance compared to just providing data to justify a publication, as similar studies claim apoptosis, pyroptosis, and other modes of cell death based on correlations with other parameters. Although these types of studies are the majority that implicate ferroptosis, the conclusions regarding ferroptosis are not rigorously justified and do not provide convincing support for ferroptosis as the major mode of cell death during APAP hepatotoxicity.

The second and very rare type of studies includes a more direct investigation of ferroptosis mechanisms in APAP-induced liver injury^[94,102,103]^. In the most prominent investigation, nearly 100% protection was reported for this model following pretreatment with ferrostatin-1, vitamin E, or deferoxamine ^[94]^. However, the experimental conditions used in this particular study were very unusual and are generally not employed in most studies. A low overdose of 200 mg/kg APAP appeared to have caused severe liver injury in fasted C57Bl/6J mice within 3 h^[94]^. This raises the question of whether some of these results are model-specific, as neither ferrostatin-1 nor proven vitamin E loading of liver membranes was shown to be protective in the regular model of liver injury at 6 h after 300 mg/kg APAP^[73,97]^. Most interesting was that the LPO parameters MDA and 12-HETE measured in this model were only increased by 75–100%^[94]^. Although these results are consistent with many studies published over the years, this degree of LPO is quantitatively insufficient to cause cell death in APAP hepatotoxicity^[73,75,94,103,104]^ and other acute liver injury models^[105]^. This conclusion is not just based on absolute numbers but also on comparison to the same parameters in positive controls for LPO^[73,75,105]^. In contrast, immunohistochemistry for 4-HNE showed extensive staining of the entire necrotic area^[94]^. However, without negative controls, i.e., staining assessment in the absence of the primary 4-HNE antibody, some of the staining could be nonspecific. Thus, this report^[94]^ is internally inconsistent and raises concerns about the conclusions that ferroptosis is a critical mode of cell death under normal and clinically relevant conditions. Unfortunately, no follow-up studies on APAP hepatotoxicity were published by this group. Most other mechanistic reports on ferroptosis in the APAP model use only ferrostatin-1^[102,106]^, whose efficacy in vivo has been questioned^[97,107]^. One caveat may need to be considered. In studies where the ferroptosis inhibitor and APAP are injected intraperitoneally at the same time, there is a chance that the inhibitor is diluted, and there could be reduced absorption and diminished efficacy of the drug. A way around this would be to either use different routes of administration (intravenous) or pretreatment to allow absorption of the drug before APAP injection. A more potent in vivo ferroptosis inhibitor, UAMC3203^[107]^, also showed excellent protection against APAP toxicity in the presence of limited LPO^[103]^, but this compound also protected in the absence of LPO through off-target effects^[97]^. Thus, there is very little credible and reproducible evidence to support the conclusion that ferroptosis is a relevant mode of cell death during APAP hepatotoxicity under normal conditions.

### 5.3 Acetaminophen-induced ferroptosis and exogenous iron

When mice are treated with a combination of a moderate amount of ferrous iron and an APAP overdose, the injury is dramatically aggravated^[75]^. Under these conditions, there is a significant increase in protein nitration, but more importantly, there is now also extensive LPO^[75]^. Not unexpectedly, an iron chelator still protects, but inhibitors targeted against the formation of mitochondrial peroxynitrite or the enhanced scavenging of peroxynitrite are now ineffective^[75,81]^. In addition, a ferroptosis inhibitor now reduces the injury^[97]^. Together, these data are consistent with the concept of ferroptosis under these conditions. However, what is less recognized is that the original erastin-mediated ferroptosis induced a mitochondria-independent oxidant stress, causing cell death, did not trigger cellular ATP depletion, and the morphology of the mitochondria indicates that they are smaller and denser, not swollen as is generally observed during necrotic cell death^[13]^. These features are inconsistent with APAP-induced cell death, which involves mitochondrial swelling, and the entire cell death mechanism is critically dependent on mitochondrial-derived oxidant stress, mitochondrial dysfunction with the MPTP opening, mitochondrial matrix swelling, and rupture of the outer membrane^[5,108,109]^. Therefore, because the APAP+Fe^2+^-induced cell death meets most but not all criteria of ferroptosis, it is more appropriate to consider this a ferroptosis-like cell death.

This raises the question of whether some specific parameters or biomarkers can define and distinguish ferroptosis and ferroptosis-like cell death. As was pointed out in a recent editorial, ferroptosis requires iron, driving a Fenton reaction-mediated lipid peroxidation that ruptures the cell membrane^[101]^. However, the susceptibility to ferroptosis is determined by many antioxidant defense systems in the cell, but none of these parameters can be a definitive marker for ferroptosis^[101]^. Based on these ferroptosis characteristics, we concluded that the cell death induced by Fe+APAP, but not APAP alone, met most criteria for ferroptosis. Because the original definition of ferroptosis did not include mitochondrial dysfunction and ATP depletion^[13]^, which is a hallmark of APAP-induced cell death with or without LPO, we decided to call it ferroptosis-like cell death to distinguish it from the original definition. However, this is a personal choice to describe a process that is closely related to ferroptosis but also has some relevant differences.

## 6. Clinical Implications of the Modes of APAP-Induced Cell Death

The review of the literature demonstrates that APAP induces necrosis that is driven by a mitochondrial dysfunction-centered cell death. This is the main reason why the clinical antidote N-acetylcysteine, which supports hepatic GSH synthesis^[110]^, is highly effective in protecting the liver against APAP hepatotoxicity by scavenging NAPQI and peroxynitrite^[36]^. More recently, fomepizole (4-methylpyrazole) came into focus as another effective antidote currently in clinical trials due to its inhibitory effect on Cyp2E1 and JNK^[111,112]^. Under these normal clinical conditions, there is no evidence for relevant LPO and ferroptotic cell death in patients. The literature review also indicates that changes in fatty acid composition and vitamin E levels, as well as a moderate dose of ferric iron, can dramatically change the mode of cell death, with LPO now being the dominant mechanism. However, in Western countries, vitamin E deficiency is rare, but taking iron supplements is relatively common. Co-ingestion of ferrous iron with an overdose of APAP can seriously accelerate and aggravate the injury mechanism in humans^[82,83]^. It is important to recognize that this requires co-ingestion of the iron with an APAP overdose; it does not make a person who takes iron supplements with therapeutic doses of APAP susceptible to hepatotoxicity. Nevertheless, if there is co-ingestion of iron with an APAP overdose, the standard antidotes of APAP are only partially effective^[81]^. For early presenting patients, it can be expected that fomepizole and NAC are still useful in limiting the injury through Cyp2E1 inhibition and NAPQI scavenging, respectively. But for patients presenting to the clinic when drug metabolism is advanced or completed, these drugs will no longer provide any benefit^[81]^. Under these conditions, treatment with clinically approved iron chelators may currently be the only therapeutic option. In the future, fast-acting and potent lipid peroxidation inhibitors may be needed for this rare clinical scenario.

## 7. Summary and Conclusions

Elucidating the mode of cell death is critical for the identification of new therapeutic targets and the development of new antidotes against drug-induced liver injury. The mechanisms of APAP hepatotoxicity have been studied extensively during the last decades, and key cell death signaling events were discovered^[5]^. However, as previously reviewed, despite some overlap between signaling events in APAP-induced cell death and apoptosis^[113]^, necroptosis^[53,114]^, and pyroptosis^[115]^, essential features of these cell death mechanisms are clearly missing in APAP toxicity. In contrast, at least superficially, there are many similarities between ferroptosis and APAP-induced necrosis, including GSH depletion, oxidant stress, the role of iron, and at least some LPO. However, a more detailed assessment of these mechanisms reveals fundamental differences that argue against ferroptosis. These include that GSH depletion does not inhibit GPx4 activity, mitochondrial superoxide is the precursor of the actual toxin peroxynitrite, iron is an important catalyst for protein nitration, and LPO is quantitatively not a relevant event. Thus, under normal experimental and clinically relevant conditions, APAP overdose-induced liver injury does not involve ferroptosis. Hence, antidotes such as N-acetylcysteine (scavenger of NAPQI and peroxynitrite) and fomepizole (inhibitor of Cyp2E1 and JNK) can be highly effective^[4]^. The iron chelator desferal was shown to be protective as pretreatment^[75,103]^ but also as posttreatment 15 min after APAP^[103]^. Nevertheless, because most patients seek medical attention 4–24 hours after the overdose, the therapeutic window of iron chelator treatment requires further investigation^[75]^. In striking contrast to these normal conditions, the co-administration of a sub-therapeutic dose of ferrous iron with the APAP overdose totally changed the mechanism of cell death, with LPO now as the main injury event, which suggests that it is now a ferroptosis-like mechanism.

What can be learned from the experience with APAP hepatotoxicity and acute liver failure? First, it is critical to focus on investigating detailed mechanisms of a clinically relevant model and not just draw conclusions about cell death modes based on one or more individual parameters, e.g., GPx4 protein expression, MDA levels, etc. If one talks about mechanisms, this should include assessment of the exact reactive oxygen species involved, the sources that generate the ROS, the cellular targets that are affected, and the detailed pathophysiological consequences of this stress. This avoids superficial and erroneous mechanistic conclusions that lack any clinical relevance and prevent the assessment of the impact of therapeutic targets or the interventions that are being investigated.
